# Supplementary material for: A virus-like particle-based bivalent PCSK9 vaccine lowers LDL-cholesterol levels in non-human primates
Source: NPJ Vaccines. 2023 Sep 28;8:142. doi: 10.1038/s41541-023-00743-6 (PMC10539315; doi:10.1038/s41541-023-00743-6)
Supplement: Supplementary file 1 — Supplemental Material [file 41541_2023_743_MOESM1_ESM.pdf]

Supplementary Information

Supplementary Table 1

Baseline characteristics of NHPs enrolled in this study[1]

ID	Total Cholesterol (mg/dL)	Triglycerides (mg/dL)	Sex	Weight (kg)	Age (years)
1-A	174.7	56.8	F	10.25	8.5
1-B	160.1	98.0	M	16.14	8.5
1-C	151.9	30.1	F	6.09	6.3
1-D	149.9	81.3	F	10.56	10.5
1-E	147.4	62.4	M	12.1	7.3
1-F	135.3	102.5	F	10.58	7.3
1-G	132.2	60.1	M	14.29	7
1-H	130.3	25.6	F	6.83	10.5
Group 1 means	147.1	60.8	3M, 5F	10.3	8.1
2-A	165.8	105.8	M	18.64	7.5
2-B	155.0	31.2	F	8.31	7.3
2-C	130.3	60.1	M	13.34	7.5
2-D	134.7	81.3	F	7.85	8.5
2-E	139.1	83.5	M	12.23	6.5
2-F	140.4	42.3	F	6.25	5.5
2-G	143.6	25.6	F	6.16	8.3
2-H	151.9	53.5	F	8.67	12.3
Group 2 means	145.9	67.4	3M, 5F	10.7	8.2
3-A	153.1	55.7	F	5.75	7
3-B	163.3	127.0	M	14.3	7.5
3-C	145.5	93.5	F	8.71	12.5
3-D	141.7	32.3	M	11.36	8.3
3-E	166.5	35.6	F	10.23	8.5
3-F	131.5	143.7	F	10.59	10.5
3-G[2]	138.5	42.3	M	9.96	10.5
3-H	132.2	31.2	M	13.34	7.5
Group 3 means	146.4	67.0	4M, 4F	10.6	9.0

[1] There were no statistically significant differences in baseline markers between groups.
[2] This macaque was withdrawn from the study.

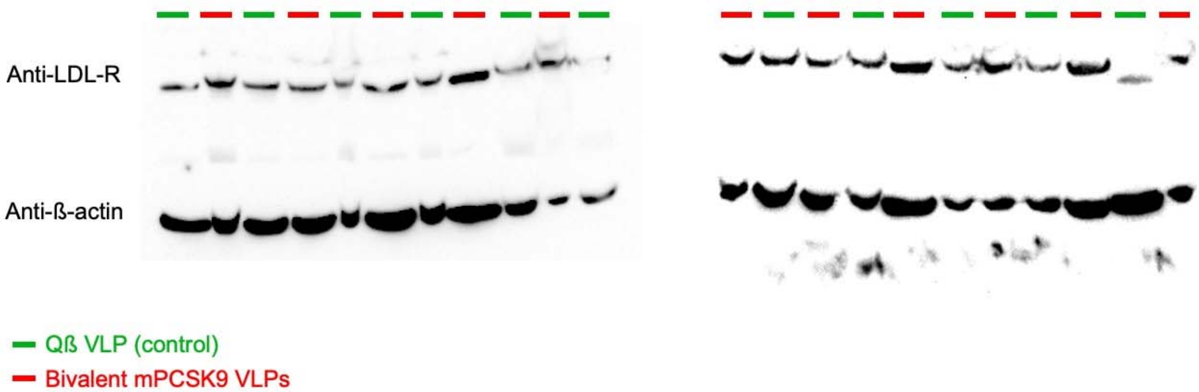

Supplementary Figure 1. Western blot data graphed in Fig. 4. Liver expression of LDL-R and ß-actin were measured by Western blot, samples obtained from mice immunized with Qß VLPs (lanes denoted with green bars) or bivalent mPCSK9-VLPs (red bars)

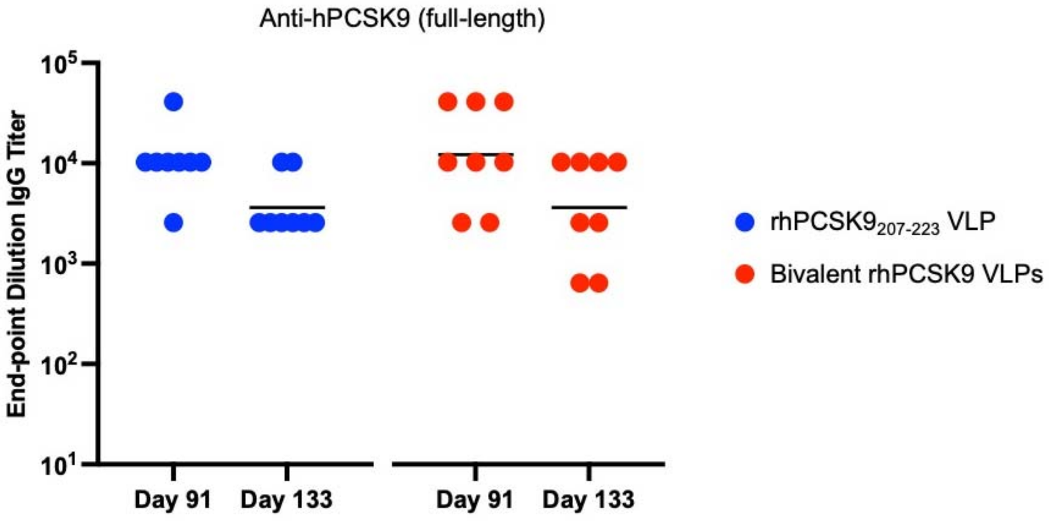

Supplementary Figure 2. Longevity of anti-PCSK9 antibody responses in immunized rhesus macaques. Plasma was obtained at day 91 and day 133 of the study. Endpoint dilution IgG titers against full-length hPCSK9 were measured by ELISA. Each data point represents an individual macaque and the geometric mean titer for each group is shown using a black line.

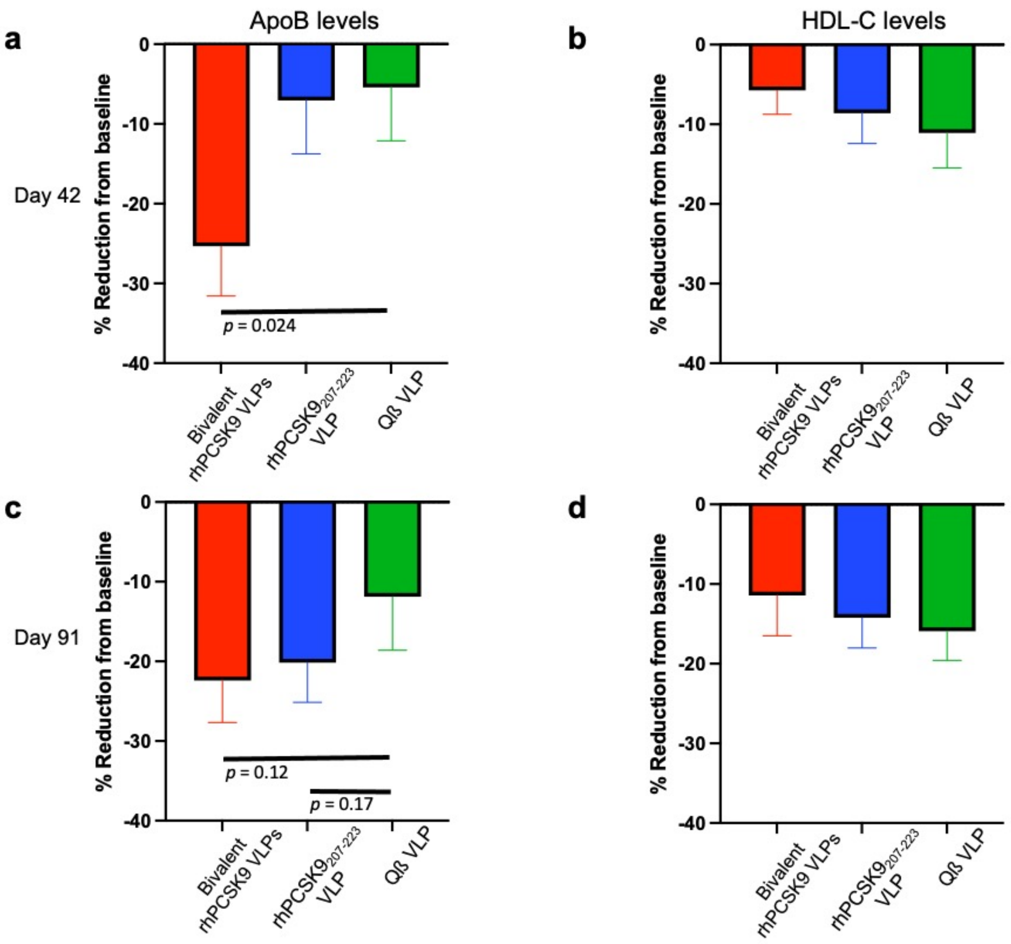

Supplementary Figure 3. Plasma ApoB and HDL-C levels in immunized rhesus macaques. Plasma was obtained after two immunizations (day 42, **a** and **b**) and two weeks after daily simvastatin administration began (day 91, **c** and **d**). Plasma ApoB (**a** and **c**) and HDL-C (**b** and **d**) levels were measured and compared to baseline (at d0, prior to prime). Bars represent mean % reduction from baseline, error bars show SEM. Significance was determined by one-tailed t test.

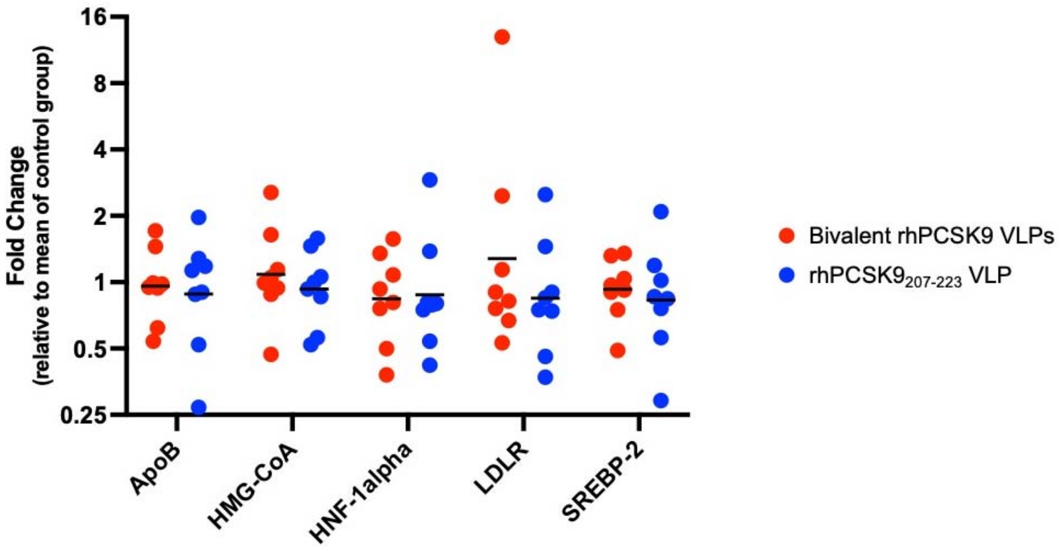

Supplementary Figure 4. Relative mRNA expression in vaccinated groups of rhesus macaques. Liver mRNA levels were determined by qRT-PCR. Cycle threshold (Ct) values were determined for each mRNA species, normalized using a housekeeping mRNA (Actin) to determine a delta CT (dCT) value, and then compared to the mean control dCT value from the group immunized with Qß VLPs, to determine the fold change of mRNA expression.

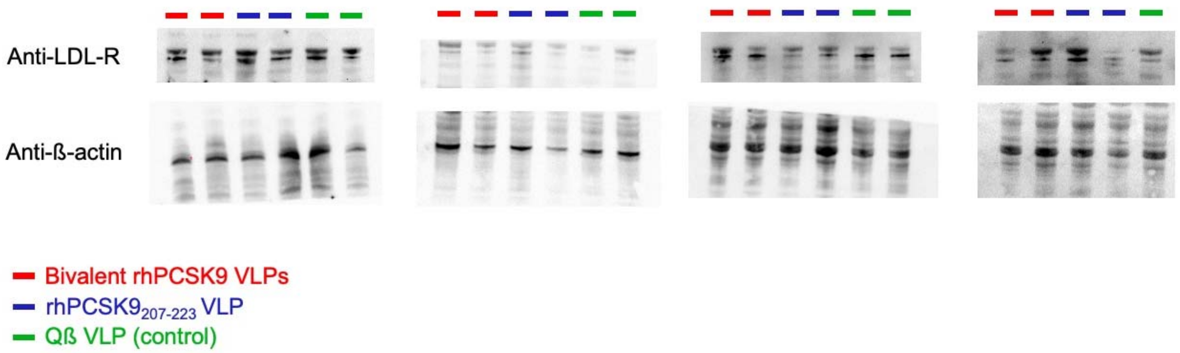

Supplementary Figure 5. Western blot data graphed in Fig. 9d. Liver expression of LDL-R and ß-actin were measured by Western blot, samples obtained from rhesus macaques immunized with bivalent rhPCSK9-VLPs (lanes denoted with red bars), rhPCSK9$_{207\text{-}223}$-VLPs (blue bars), or Qß VLPs (green bars).
